# Mechanistic actions of eldecalcitol and alfacalcidol across the muscle-metabolic axis: A selectivity perspective in sarcopenia and type 2 diabetes

**DOI:** 10.1016/j.isci.2026.115945

**Published:** 2026-04-30

**Authors:** Maria Teresa Cambria, Mariachiara Campanella, Cristina Russo, Aleksandra Agafonova, Sofia Surdo, Maria Stella Valle, Lucia Malaguarnera

**Affiliations:** 1 Department of Biomedical and Biotechnological Sciences, Section of Medical Biochemistry, University of Catania, Catania 95123, Italy; 2 Department of Biomedical and Biotechnological Sciences, Section of Clinical Pathology, University of Catania, Catania 95123, Italy; 3 Italian Center for the Study of Osteopathy (CSDOI), Catania 95124, Italy; 4 Department of Biomedical and Biotechnological Sciences, Section of Physiology, University of Catania, Catania 95123, Italy

**Keywords:** Health sciences

## Abstract

Sarcopenia and type 2 diabetes mellitus (T2DM) share a convergent molecular landscape characterized by chronic low-grade inflammation, oxidative stress, mitochondrial dysfunction, and impaired insulin-anabolic signaling in skeletal muscle. Although vitamin D receptor (VDR) activation has been implicated in modulating each of these domains, vitamin D analogs are frequently regarded as pharmacologically interchangeable. However, it remains unclear whether structurally distinct VDR ligands may exhibit functional selectivity, generating qualitatively divergent transcriptional and signaling outcomes within the muscle-metabolic axis. In this review, we examine eldecalcitol and alfacalcidol, two clinically approved active vitamin D analogs, as pharmacological models to interrogate ligand-specific VDR signaling. We analyze differences in structural configuration, binding protein affinity, metabolic activation, and receptor engagement dynamics, and evaluate their intersection with three key domains relevant to sarcopenia and T2DM: NF-κB-mediated inflammation, Nrf2-dependent redox regulation, and PI3K/Akt/mTOR-FOXO signaling governing muscle protein turnover. We stratify available evidence into direct skeletal muscle data, systemic and non-muscle findings, and hypothesis-driven integrative models. While preclinical studies suggest context-dependent modulation of inflammatory and anabolic pathways, direct comparative analyses demonstrating ligand-specific VDR transcriptional bias in human skeletal muscle remain lacking. Accordingly, the concept of functional selectivity is presented as a biologically plausible but as yet unproven framework, intended to guide future translational investigations.

## Graphical abstract

**Figure:**
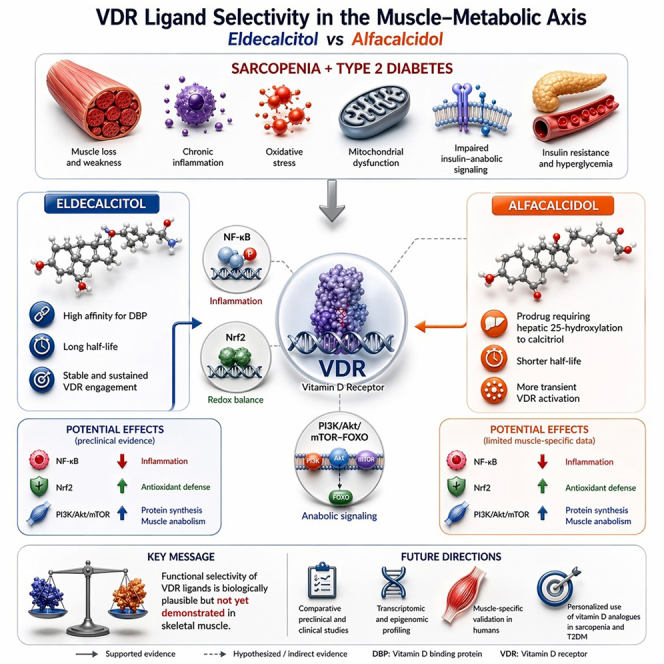

## Introduction

Sarcopenia is a progressive and generalized skeletal muscle disorder characterized by declines in muscle mass, strength, and physical performance and is increasingly recognized as a clinically relevant complication of type 2 diabetes mellitus (T2DM).^1^ The coexistence of these conditions establishes a bidirectional pathological loop in which insulin resistance, chronic hyperglycemia, and metabolic inflammation accelerate muscle degradation, while sarcopenia further exacerbates glucose intolerance and metabolic dysfunction.^2^ As a result, individuals affected by both disorders experience increased frailty, reduced mobility, higher risk of falls and fractures, and worsened clinical outcomes. At the molecular level, sarcopenia and T2DM share overlapping pathophysiological features, including chronic low-grade inflammation, elevated oxidative stress, mitochondrial dysfunction, and impaired insulin-anabolic signaling within skeletal muscle.^2^^,^^3^^,^^4^ Accumulation of reactive oxygen species (ROS) increased production of pro-inflammatory cytokines such as tumor necrosis factor-α (TNF-α) and interleukin-6 (IL-6), and dysregulation of redox-sensitive signaling pathways contributes to muscle atrophy, defective regeneration, and insulin resistance.^5^^,^^6^ These shared mechanisms suggest that therapeutic strategies targeting inflammation, oxidative stress, and anabolic resistance may exert dual benefits on muscle integrity and metabolic homeostasis. Vitamin D and its analogs have traditionally been employed for the prevention and treatment of bone and mineral disorders.^7^ Beyond its classical role in calcium-phosphate homeostasis, activation of the vitamin D receptor (VDR) regulates immune responses, redox balance, mitochondrial dynamics, and metabolic gene expression.^8^^,^^9^ Experimental studies suggest that VDR activation may suppress NF-κB-mediated inflammatory pathways, enhance Nrf2-dependent antioxidant defenses, and influence insulin-anabolic signaling cascades.^10^ Despite these insights, vitamin D and its analogs are frequently treated as pharmacologically interchangeable, implicitly assuming that VDR activation represents a uniform biological signal irrespective of ligand structure or pharmacokinetic behavior.^11^ This assumption may be overly reductive. In receptor pharmacology, the concept of functional selectivity, also referred to as ligand bias, describes the possibility that structurally distinct ligands acting on the same receptor stabilize different receptor conformations, thereby inducing qualitatively distinct transcriptional programs or downstream signaling outputs.^12^ While this framework is increasingly recognized across receptor biology, its application to VDR signaling in skeletal muscle and metabolic disease remains largely conceptual. Within this conceptual framework, active vitamin D analogs such as eldecalcitol and alfacalcidol offer clinically relevant pharmacological models with which to explore this possibility.

Eldecalcitol, a synthetic derivative of 1,25-dihydroxyvitamin D_3_, exhibits enhanced binding affinity for vitamin D-binding protein and prolonged systemic exposure, resulting in sustained VDR activation.^13^^,^^14^ In contrast, alfacalcidol is a prodrug requiring hepatic 25-hydroxylation to generate calcitriol, leading to distinct pharmacokinetic dynamics and temporal exposure profiles. It increases circulating calcitriol levels and has been widely used in elderly populations and patients with impaired renal function.^15^ Although both compounds ultimately activate VDR, established differences in ligand structure, receptor engagement kinetics, and tissue exposure raise the theoretical possibility that they may induce differential transcriptional integration across inflammatory, redox, and anabolic signaling domains. Whether such ligand-specific effects occur in skeletal muscle and whether they are biologically meaningful in the context of sarcopenia and T2DM remains unresolved. Current mechanistic evidence is predominantly derived from preclinical systems, often outside skeletal muscle, and direct head-to-head comparative studies are scarce. Moreover, skeletal muscle-specific transcriptional profiling following treatment with distinct VDR ligands remains limited in humans. Consequently, it is unclear whether reported anti-inflammatory, antioxidant, or anti-catabolic effects reflect generalized VDR activation, systemic endocrine modulation, or true ligand-specific signaling integration. Despite its conceptual appeal, the application of functional selectivity to VDR biology in metabolic muscle disorders remains largely hypothetical. Demonstrating true ligand bias would require comparative analyses of VDR-dependent transcriptional programs, co-regulator recruitment dynamics, chromatin occupancy, and pathway-specific signaling outputs under controlled experimental conditions, approaches that are currently underexplored in sarcopenia and T2DM research.

Accordingly, the aim of this review is 2-fold: first, to synthesize current evidence linking VDR signaling to inflammatory, redox, mitochondrial, and anabolic pathways relevant to sarcopenia and T2DM; second, to evaluate whether functional selectivity offers a useful, albeit still unproven, framework for interpreting potential ligand-specific differences between active vitamin D analogs. Importantly, this framework is presented as a hypothesis-generating tool rather than an established mechanistic paradigm. Throughout the manuscript, established findings are distinguished from hypothesis-based interpretations to avoid overextension of the currently available evidence.

## Pathophysiology of sarcopenia and T2DM

The following section summarizes current mechanistic evidence across inflammatory, redox, and anabolic pathways. While these pathways are well established in the context of muscle and metabolic regulation, their interpretation within a ligand-specific VDR framework remains speculative and should be considered with caution.

The incidence and pathophysiology of sarcopenia are complex and influenced by a combination of intrinsic factors (e.g., age, ethnicity, and lifestyle) and extrinsic factors (e.g., socioeconomic status and country of residence). Etiologically, sarcopenia can be divided into two categories: primary and secondary forms. Primary sarcopenia is attributed exclusively to the aging process,^16^ whereas secondary sarcopenia results from chronic systemic conditions or inflammatory processes, such as malignancies or organ failure. Additional risk factors include a sedentary lifestyle, physical inactivity, malabsorption, and insufficient protein intake.^17^ In 2018, the European Working Group on Sarcopenia in Older People (EWGSOP2) updated its diagnostic and treatment criteria, introducing new subtypes of sarcopenia: acute, in which symptoms persist for less than six months, and chronic sarcopenia, which refers to symptoms lasting longer than six months, often correlating with increased mortality.^18^

**Figure 1:**
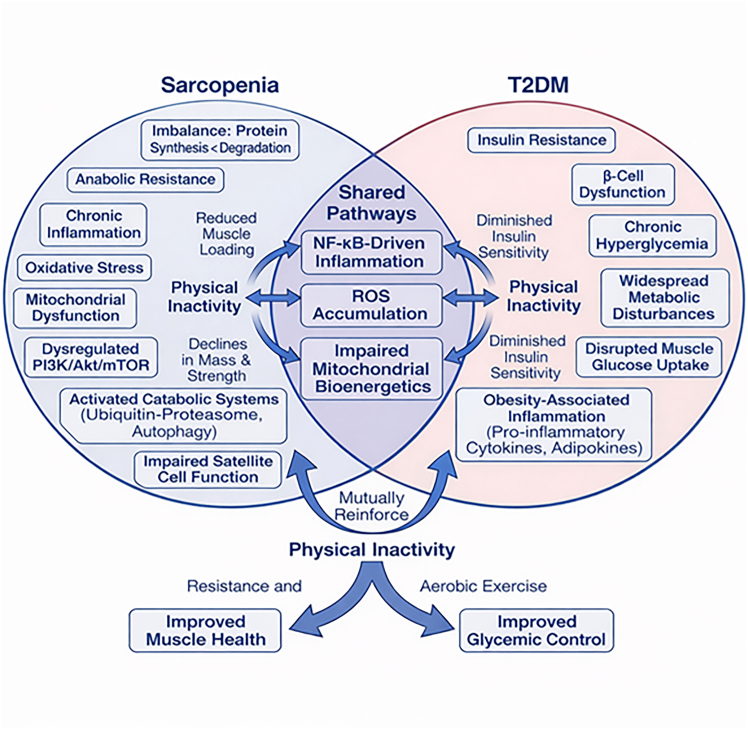
Pathophysiological interplay between sarcopenia and T2DM Both conditions converge on shared molecular mechanisms, including NF-κB-driven chronic inflammation, increased ROS, mitochondrial dysfunction, and impaired bioenergetics. Alterations in the PI3K/Akt/mTOR signaling pathway contribute to reduced protein synthesis, anabolic resistance, and defective glucose uptake. Physical inactivity acts as a central amplifying factor, whereas strength and aerobic exercise represent effective interventions to improve skeletal muscle integrity and glycemic regulation. This figure summarizes established pathophysiological mechanisms and does not imply ligand-specific VDR effects.

Therefore, early identification and intervention are crucial to prevent or delay disease progression. Skeletal muscle consists of structural and contractile proteins, so any reduction in protein content directly compromises muscle integrity and function. Muscle protein synthesis is regulated by several signaling pathways, particularly the PI3K/AKT/mTOR pathway, which becomes dysregulated with aging, chronic inflammation, and anabolic resistance. Sarcopenia arises from an imbalance between muscle protein synthesis and degradation, driven by age-related anabolic resistance, chronic inflammation, oxidative stress, and mitochondrial dysfunction (Figure 1).^5^ Dysregulation of the PI3K/Akt/mTOR pathway reduces protein synthesis, while the activation of catabolic systems, including the ubiquitin-proteasome and autophagy-lysosome pathways, accelerates muscle breakdown.^19^^,^^20^ These alterations are compounded by impaired satellite cell function and reduced regenerative capacity. T2DM is characterized by insulin resistance and progressive β-cell dysfunction, leading to chronic hyperglycemia and widespread metabolic disturbances.^21^^,^^22^ In skeletal muscle, insulin resistance disrupts glucose uptake and anabolic signaling, directly linking metabolic dysfunction to muscle atrophy. Obesity-associated inflammation further exacerbates this process through increased secretion of pro-inflammatory cytokines and adipokines, reinforcing insulin resistance and muscle catabolism. Importantly, sarcopenia and T2DM converge on shared molecular pathways, including NF-κB-driven inflammation, ROS accumulation, and impaired mitochondrial bioenergetics.^4^^,^^23^^,^^24^ Physical inactivity is a major modifiable factor contributing to the development and progression of sarcopenia and T2DM. Reduced muscle loading leads to declines in muscle mass and strength, while also diminishing insulin sensitivity. Evidence from interventional studies indicates that physical inactivity exacerbates these shared pathophysiological mechanisms, whereas resistance and aerobic exercise improve both muscle health and glycemic control.^4^^,^^25^^,^^26^ Collectively, these observations support the concept of a pathophysiological continuum in which metabolic and muscular dysfunction mutually reinforce each other through interconnected inflammatory, redox, and anabolic signaling networks (Figure 1).

## Inflammatory pathways and chronic low-grade inflammation

Chronic low-grade inflammation represents a central feature linking sarcopenia and T2DM. Persistent activation of NF-κB signaling promotes the transcription of pro-inflammatory cytokines, such as TNF-α and IL-6, which impair insulin signaling and stimulate muscle proteolysis.^4^^,^^23^^,^^27^ TNF-α interferes with insulin receptor substrate (IRS) signaling through serine phosphorylation, reducing PI3K/Akt activation and contributing to insulin resistance.^3^^,^^6^ Systemic inflammatory markers, including C-reactive protein, correlate with reduced muscle strength and metabolic dysfunction, highlighting the systemic nature of inflammatory crosstalk between muscle and metabolic tissues.^1^^,^^27^ In skeletal muscle, sustained NF-κB activation contributes to the transcriptional upregulation of catabolic pathways and the suppression of anabolic signaling, thereby promoting muscle atrophy and metabolic impairment.^28^ This inflammatory milieu promotes a self-perpetuating cycle of tissue damage and metabolic deterioration.^4^^,^^23^ Experimental evidence supports a role for VDR signaling in modulating inflammatory responses, including the inhibition of NF-κB activation through interaction with upstream regulatory components such as IκB kinase β.^10^ These findings suggest that VDR activation may attenuate inflammation-associated muscle catabolism and insulin resistance. However, most available mechanistic evidence derives from *in vitro* systems or non-muscle tissues, and direct confirmation of these effects in human skeletal muscle remains limited. Furthermore, no studies have directly compared the effects of distinct VDR ligands on NF-κB signaling in skeletal muscle.

Inflammation and oxidative stress are tightly interconnected in both sarcopenia and T2DM. Excessive ROS production impairs mitochondrial function, damages cellular macromolecules, and activates redox-sensitive inflammatory pathways.^26^^,^^29^ In aging and diabetic muscle, diminished antioxidant capacity exacerbates oxidative damage, contributing to muscle weakness and insulin resistance.^30^

The transcription factor Nrf2 regulates cellular antioxidant defenses by inducing enzymes such as superoxide dismutase, catalase, and glutathione peroxidase.^31^^,^^32^ Under physiological conditions, Nrf2 activation maintains redox homeostasis and protects against oxidative injury, whereas its suppression in sarcopenia and T2DM contributes to impaired antioxidant responses and increased cellular stress.^5^^,^^9^ Vitamin D signaling has been proposed to influence redox balance, potentially through the modulation of Nrf2-dependent pathways. Experimental observations suggest that VDR activation may enhance antioxidant defenses; however, these findings are predominantly derived from non-muscle models or indirect systems. Accordingly, whether VDR signaling directly regulates Nrf2 activity in skeletal muscle, and whether structurally distinct VDR ligands differentially modulate redox homeostasis, remains unclear. These interpretations should therefore be considered within a hypothesis-generating framework rather than as evidence of ligand-specific effects.

## Vitamin D analogs: alfacalcidol and eldecalcitol

Alfacalcidol (1α-hydroxyvitamin D_3_) is a synthetic prodrug of calcitriol that lacks the 25-hydroxyl group. Unlike vitamin D, it is already hydroxylated at the 1α position, and thus does not require the renal enzyme 1α-hydroxylase. After administration, alfacalcidol is rapidly metabolized in the liver to calcitriol, resulting in a gradual increase in circulating calcitriol levels. Calcitriol is then catabolized in the kidney to 1,24(R),25-trihydroxyvitamin D_3_ by 24-hydroxylase (CYP24A1).^33^ Further therapeutic improvement led to eldecalcitol development (1,25-dihydroxy-2-(3-hydroxypropoxy) vitamin D_3_).^34^^,^^35^ Unlike alfacalcidol, eldecalcitol contains a 2β-position hydroxypropyloxy group, conferring greater metabolic stability and selective bone effects Figure 1). Over 30 years of clinical experience demonstrate both efficacy and safety for these analogs.^36^ Eldecalcitol is more frequently described than calcitriol because it exhibits a higher binding affinity for vitamin D binding protein (DBP).^37^ Consequently, eldecalcitol has a longer half-life (53 h) in humans.^38^ X-ray crystallography reveals that eldecalcitol’s 3-hydroxypropoxy group forms three hydrogen bonds with DBP: the terminal hydroxyl interacts with Pro87 backbone carbonyl and Ser79 side chain hydroxyl, while the ether oxygen creates a third bond with Ser79 hydroxyl.^39^ Alfacalcidol demonstrates intermediate pharmacokinetics with a 47-h half-life, permitting once-daily dosing compared to calcitriol’s typical twice-daily requirement.^15^ Structural analyses using NMR and X-ray crystallography reveal nearly identical calcitriol-VDR and eldecalcitol-VDR complexes, except for 3-hydroxypropoxy interactions.^12^^,^^40^ In eldecalcitol, the terminal hydroxyl forms bifurcated hydrogen bonds with Arg278 guanidino group and Thr142 main chain carbonyl, while the alkyl chain establishes CH-π interactions with Tyr143. This 3-hydroxypropoxy group occupies an additional VDR binding pocket, stabilizing the complex and providing an extended residence time as well as improved binding affinity.^41^

Vitamin D analogs have been developed to enhance VDR activation while minimizing calcemic side effects.^7^ Alfacalcidol and eldecalcitol differ in their metabolic activation, binding properties, and tissue exposure, which may influence their biological actions beyond bone.^42^ Structural and pharmacokinetic analyses indicate that eldecalcitol exhibits prolonged systemic persistence and enhanced VDR engagement, whereas alfacalcidol acts indirectly via conversion to calcitriol.^14^

**Figure 2:**
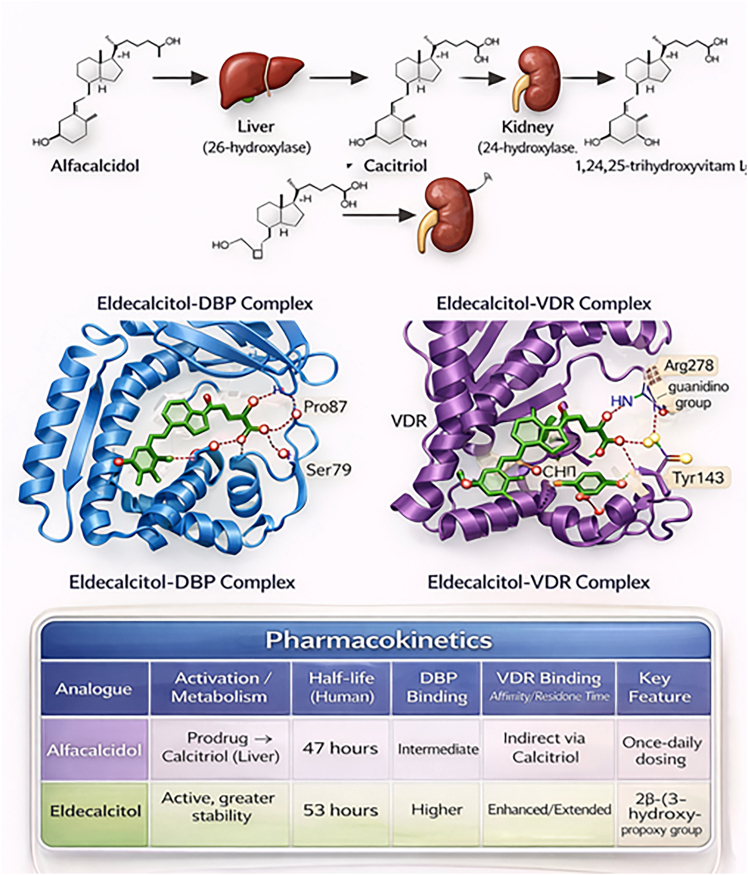
Differences in metabolic activation, binding affinity, half-life, and receptor engagement between the two compounds Metabolic activation, molecular interactions, and pharmacokinetic properties of the vitamin D analogues alfacalcidol and eldecalcitol. DBP; VDR. 2β-(3-hydroxypropoxy) denotes a structural side chain unique to eldecalcitol that enhances receptor affinity and metabolic stability. Arg (arginine), Tyr (tyrosine), Pro (proline), and Ser (serine) indicate amino acid residues involved in ligand-protein interactions within the DBP and VDR binding pockets. The structural and pharmacokinetic differences illustrated are supported by experimental evidence; however, their potential impact on downstream signaling and muscle-specific VDR responses remains incompletely defined.

These differences provide a rationale for comparing their mechanistic effects in muscle and metabolic tissues (Figure 2).^43^

## Mechanistic actions of eldecalcitol and alfacalcidol in sarcopenia and T2DM in the context of functional selectivity

In light of the functional selectivity framework outlined above, the biological actions of active vitamin D analogs in sarcopenia and T2DM can be examined across three interrelated signaling domains: inflammatory regulation, redox homeostasis, and insulin-anabolic signaling. These domains are highly interconnected and should be interpreted as components of an integrated regulatory network rather than as independent pathways.

Importantly, the strength and tissue specificity of the available evidence differ substantially across these domains. To improve clarity and address the heterogeneity of available data, the evidence was structured using an evidence-stratified approach. Specifically, findings were categorized according to experimental system and degree of skeletal muscle validation, distinguishing direct muscle-specific evidence from systemic and translational observations. Given the diversity of models and study designs, the reported molecular effects should not be interpreted as evidence of ligand-specific VDR functional selectivity. Moreover, no head-to-head comparative studies have directly evaluated transcriptional bias or differential VDR-dependent signaling integration between eldecalcitol and alfacalcidol in skeletal muscle. Accordingly, throughout this section, established findings are presented alongside hypothesis-based interpretations, with particular attention to the level of experimental support and tissue specificity. A structured summary of this approach is provided in Table 1.

**Table 1:** Evidence-stratified summary of VDR-related molecular mechanisms and functional outcomes across experimental systems in sarcopenia and type 2 diabetes mellitus (T2DM)

Experimental System	Study Type	Biological Context	Molecular Pathway	Functional Outcome	Evidence Level	Reference
Skeletal muscle (*in vitro*)	Preclinical	Muscle atrophy	PI3K/Akt/mTOR	Increased protein synthesis	Direct skeletal muscle evidence	Vainshtein and Sandri,^19^ Kitajima et al.^20^
Skeletal muscle (animal model)	Preclinical	Sarcopenia	FOXO signaling	Reduced proteolysis	Direct skeletal muscle evidence	Zhang et al.^44^
Animal model	Preclinical	Insulin resistance	NF-κB	Reduced inflammation	Indirect/systemic evidence	Zhang et al.^45^
Animal model	Preclinical	Metabolic dysfunction	Nrf2 pathway	Improved redox balance	Indirect/systemic evidence	Huang et al.,^46^ Saad El-Din et al.^47^
Human (clinical studies)	Clinical	T2DM	Multi-pathway modulation	Improved metabolic parameters	Translational association	Kawahara et al.^48^
Human (observational)	Clinical	Sarcopenia	Vitamin D-associated pathways	Association with muscle function	Translational association	Kawahara et al.,^48^ Luo et al.^49^
Integrated interpretation	Conceptual	Muscle-metabolic axis	Multi-pathway integration	Potential ligand-specific effects	Hypothesis-generating	Matsumoto et al.^43^

NF-κB, nuclear factor kappa B; Nrf2, nuclear factor erythroid 2–related factor 2; PI3K, phosphoinositide 3-kinase; FOXO, forkhead box O; ROS, reactive oxygen species; Treg, regulatory T cell.

### Direct skeletal muscle evidence

Preclinical studies in murine models of disuse and androgen-deprivation-induced muscle atrophy indicate that eldecalcitol modulates catabolic signaling pathways within skeletal muscle.

In tail-suspension and orchiectomized mouse models, treatment has been associated with reduced NF-κB activation, decreased expression of muscle-specific ubiquitin ligases (MuRF-1 and Atrogin-1), and partial preservation of muscle mass and fiber architecture.^44^^,^^45^ These findings suggest that VDR engagement may influence the balance between PI3K/Akt/mTOR-mediated anabolic signaling and FOXO-driven catabolic programs under stress conditions.^20^^,^^44^ However, these observations are largely limited to eldecalcitol and derive from preclinical models, and their direct relevance to human skeletal muscle physiology remains uncertain. Comparable mechanistic studies directly evaluating alfacalcidol in skeletal muscle tissue are limited, and head-to-head analyses between the two compounds are lacking. Therefore, while available data provide proof-of-concept for the VDR-mediated modulation of muscle signaling pathways, they do not establish ligand-specific effects or differential signaling integration between distinct VDR agonists.

### Systemic and non-muscle mechanistic evidence

Evidence supporting Nrf2 activation, suppression of inflammasome signaling, and modulation of immune cell phenotypes derives predominantly from non-muscle cellular systems and inflammatory or diabetic animal models.^46^^,^^47^ Eldecalcitol has been reported to enhance antioxidant enzyme expression and promote regulatory T cell differentiation while favoring anti-inflammatory macrophage polarization.^38^^,^^50^ These findings suggest that VDR activation may exert systemic immunomodulatory and antioxidant effects that could indirectly influence skeletal muscle homeostasis.

Nevertheless, extrapolation from immune or fibroblast systems to skeletal muscle requires caution. Functional selectivity, if present, would be expected to manifest in tissue-specific transcriptional outputs, which have not yet been directly characterized in skeletal muscle. Accordingly, these observations should not be interpreted as evidence of ligand-specific VDR signaling, but rather as indicative of broader systemic effects that may secondarily impact muscle-metabolic function.

### Clinical and translational observations

Human studies evaluating active vitamin D analogs primarily assess functional outcomes such as muscle strength, balance, fall risk, or metabolic parameters.^51^ While some trials report improvements in muscle-related endpoints, intracellular signaling pathways, and VDR-dependent transcriptional programs are rarely interrogated in muscle biopsies.^52^ As a result, clinical observations currently lack the mechanistic resolution required to distinguish between direct skeletal muscle effects and indirect systemic influences. Consequently, clinical observations cannot currently distinguish between generalized endocrine correction, systemic anti-inflammatory effects, or ligand-specific signaling bias. Collectively, existing evidence supports the biological plausibility that VDR activation intersects with inflammatory, redox, and anabolic pathways relevant to sarcopenia and T2DM.^48^^,^^49^ However, whether structurally distinct ligands such as eldecalcitol and alfacalcidol differentially integrate these pathways in a manner consistent with functional selectivity remains experimentally unresolved.

Future investigations incorporating comparative transcriptomics, co-regulator recruitment profiling, and temporal pharmacodynamic analyses in skeletal muscle will be essential to determine whether ligand-specific VDR signaling contributes to distinct metabolic-musculoskeletal outcomes.

**Figure 3:**
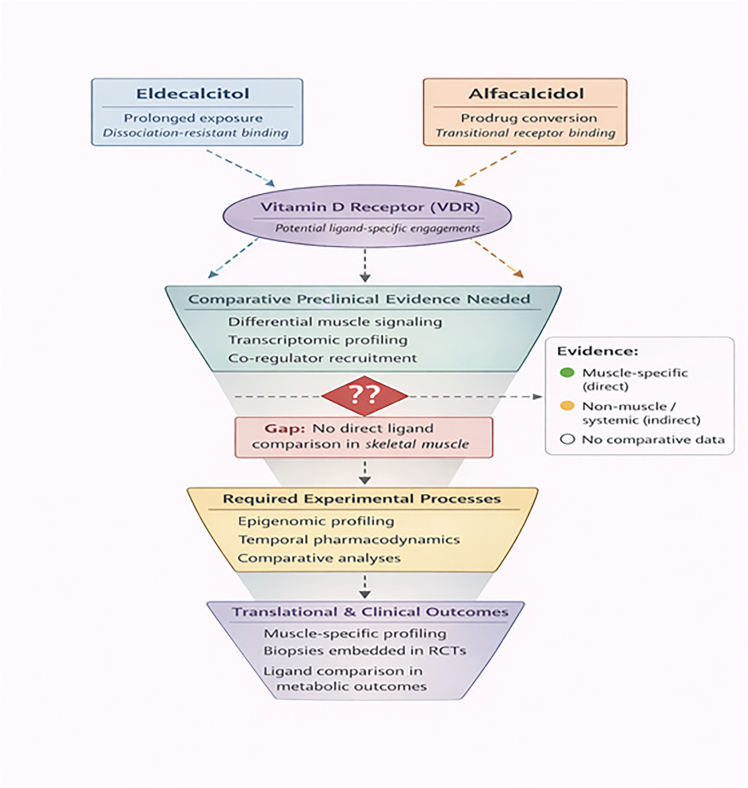
Ligand-specific engagement of the vitamin D receptor and current evidence gap in skeletal muscle Conceptual model of the interaction of two vitamin D analogues, eldecalcitol, characterized by prolonged receptor exposure and dissociation-resistant binding, and alfacalcidol, a prodrug with transient receptor engagement with the VDR. Although ligand-specific VDR signaling may influence muscle-related transcriptional programs, direct comparative evidence in skeletal muscle is lacking. Accordingly, the proposed ligand-dependent signaling effects should be interpreted as hypothesis-generating rather than as established mechanisms. The figure highlights the need for comparative preclinical studies, epigenomic and pharmacodynamic analyses, and muscle-specific profiling within clinical trials to clarify ligand-dependent effects on metabolic and musculoskeletal outcomes.

Figure 3 shows how two forms of vitamin D might act differently on the VDR. Eldecalcitol exhibits prolonged and stable binding to the VDR, whereas alfacalcidol, a prodrug, engages the receptor transiently. These ligand-specific differences may influence muscle-related transcriptional programs; however, direct evidence in skeletal muscle is lacking. Comparative preclinical and clinical studies are required to clarify their effects on metabolic and musculoskeletal analyses.

## Conclusions and future perspectives

Overall, current evidence supports a role for VDR signaling in key pathways involved in sarcopenia and T2DM, including inflammation, oxidative stress, and anabolic regulation.

Sarcopenia and T2DM represent interrelated conditions sustained by convergent inflammatory, oxidative, and metabolic disturbances within skeletal muscle. VDR signaling intersects with these domains and constitutes a biologically plausible regulatory node within the muscle-metabolic axis. Nevertheless, the extent to which structurally distinct VDR ligands exert equivalent or qualitatively divergent effects remains insufficiently clarified.

By examining eldecalcitol and alfacalcidol through the lens of functional selectivity, this review shifts the focus from the generalized question of whether VDR activation is beneficial to the more precise issue of whether distinct ligands generate differentially integrated transcriptional and signaling outputs in skeletal muscle. Although these compounds differ in structural configuration, vitamin D-binding protein affinity, and pharmacokinetic dynamics, the current body of evidence remains predominantly preclinical, heterogeneous in design, and rarely comparative. Direct skeletal muscle-specific analyses in human studies are limited, and intracellular signaling mechanisms are seldom interrogated in clinical settings (Figure 3).

At present, available data do not establish ligand-specific VDR transcriptional bias in metabolic muscle disorders. The functional selectivity framework should therefore be regarded as a structured and testable hypothesis rather than a confirmed mechanistic paradigm. The lack of head-to-head experimental designs, tissue-level transcriptomic profiling, and detailed analyses of co-regulator recruitment dynamics constrains the ability to determine whether reported biological effects reflect generalized VDR activation, indirect systemic modulation, or true ligand-dependent signaling integration.

Future progress in this field will require a transition from systemic outcome measures toward integrated molecular and translational approaches capable of resolving ligand-specific effects within skeletal muscle. Comparative transcriptomic and epigenomic profiling, detailed characterization of VDR chromatin occupancy under metabolic stress conditions, and incorporation of mechanistic endpoints into clinical studies will be essential to determine whether distinct VDR ligands differentially influence inflammatory, redox, and anabolic pathways. Such investigations would clarify whether functional selectivity represents a meaningful dimension of VDR biology in sarcopenia and T2DM or whether VDR activation operates as a broadly conserved regulatory mechanism irrespective of ligand structure.

Reframing vitamin D analog research within a functional selectivity paradigm highlights both the mechanistic promise and the evidentiary boundaries that currently define the field. The critical next step is not merely to reaffirm VDR involvement in metabolic muscle disorders, but to rigorously determine whether structurally distinct ligands differentially shape the muscle-metabolic axis under conditions of aging and metabolic stress.

## Acknowledgments

All the figures are prepared with the help of the free BioRender online tool. The authors thank the University of Catania for financial support (grant “Fondi di Ateneo 2024–2026; University of Catania, PIA.CE.RI, linea Open Access”), and PNRR: “Health Extended Alliance for Innovative Therapies, Advanced Lab-research, and Integrated Approaches of Precision” CUP: E63C22002080006.

## Author contributions

Conceptualization: M.T.C. and M.S.V.; literature research: M.C. and C.R.; writing – original draft preparation: M.T.C., M.S.V., and L.M.; graphic design: S.S.; writing-review and editing: M.C., C.R., A.A., S.S., and L.M.; project administration: M.T.C. and M.S.V.

## Declaration of interests

The authors declare no competing interests.
